# Synthesis of (*S*)-1-(2-chloroacetyl)pyrrolidine-2-carbonitrile: A key intermediate for dipeptidyl peptidase IV inhibitors

**DOI:** 10.3762/bjoc.4.20

**Published:** 2008-06-12

**Authors:** Santosh Kumar Singh, Narendra Manne, Manojit Pal

**Affiliations:** 1 New Drug Discovery, Matrix Laboratories Limited, Anrich Industrial Estate, Bollaram, Jinnaram Mandal, Medak Dist., Andhra Pradesh, India-502 325

**Keywords:** amides, DPP-IV inhibitors, L-proline, N-acylation, 2(S)-cyanopyrrolidine

## Abstract

An alternative and practical synthesis of (*S*)-1-(2-chloroacetyl)pyrrolidine-2-carbonitrile was achieved. Reaction of L-proline with chloroacetyl chloride was followed by conversion of the carboxylic acid moiety of the resulting *N*-acylated product into the carbonitrile via the corresponding amide intermediate. The synthesized pyrrolidine derivative was utilized to prepare DPP-IV inhibitor Vildagliptin.

## Background

**Figure 1:**
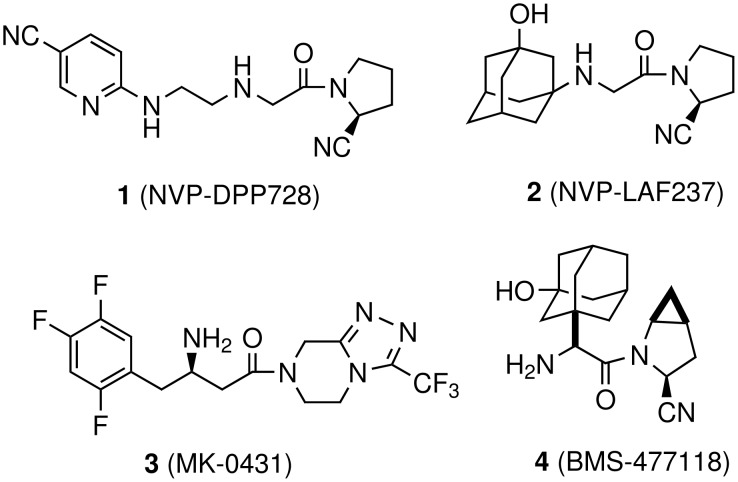
DPP-IV Inhibitors.

One of the emerging and mechanism based approaches for the treatment of type-II diabetes is dipeptidyl peptidase IV (DPP-IV; CD26; E.C. 3.4.14.5) inhibition with the help of small molecules [1–3]. DPP-IV, a member of the prolyl oligopeptidase family of serine protease, cleaves the *N*-terminal dipeptide from peptides with proline or alanine in the second position. As a result of intense pharmaceutical research, several DPP-IV inhibitors have been discovered and a few of them entered clinical development recently. These include NVP-DPP728 (**1**) [4–5], NVP-LAF237 (Vildagliptin, **2**) [6–8], MK-0431 (Sitagliptin, **3**) [9–11] and BMS-477118 (Saxagliptin, **4**) [12] (Figure 1). Vildagliptin and Sitagliptin are presently under review by US FDA as new treatment options for type-II diabetes.

The active form of glucagon-like peptide-1 (GLP-1), an incretin hormone secreted by intestinal L-cells in response to food intake [13], is a 30-amino acid peptide that stimulates insulin release, inhibits glucagon release, and slows gastric emptying. Each of these effects is beneficial in the control of glucose homeostasis in patients with type-II diabetes. However, in the presence of plasma DPP-IV the active form of GLP-1 is inactivated rapidly (t_1/2_~1 min) due to the cleavage of a dipeptide from the *N*-terminus [14–15]. Thus inhibition of DPP-IV extends the half-life of endogenously secreted GLP-1, which in turn enhances insulin secretion and improves the glucose tolerance. DPP-IV inhibitors offer several potential advantages over existing therapies including decreased risk of hypoglycemia, potential for weight loss, and the potential for regeneration and differentiation of pancreatic β-cells [1].

**Figure 2:**
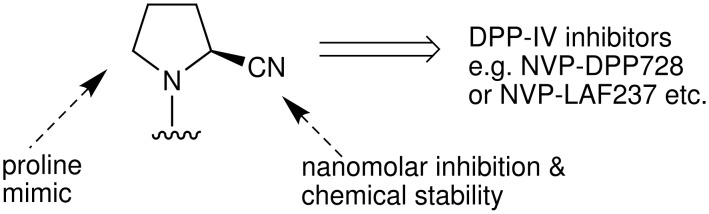
Role of 2(*S*)-cyanopyrrolidine moiety in DPP-IV inhibition.

Because of its key role in DPP-IV inhibition the 2(*S*)-cyanopyrrolidine moiety has been found to be an integral part of many DPP-IV inhibitors (Figure 1). Apart from behaving as a proline mimic, the presence of the nitrile on the five-membered ring provides (i) reversible and nanomolar inhibition of DPP-IV and (ii) chemical stability adequate for oral administration [6] (Figure 2).

**Scheme 1:**
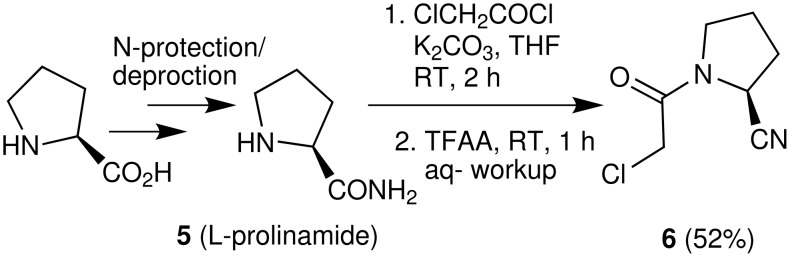
Earlier route to (*S*)-1-(2-chloroacetyl)pyrrolidine-2-carbonitrile (**6**).

Chemically, incorporation of a 2(*S*)-cyanopyrrolidine moiety into a molecule can be carried out by using (*S*)-1-(2-chloroacetyl)pyrrolidine-2-carbonitrile (**6**) as a reactant. Thus, compound **6** has become a widely used key intermediate for the synthesis of many DPP-IV inhibitors including NVP-LAF237 (**2**) that are presently under various stages of clinical evaluation [16–23]. For the development of novel DPP-IV inhibitors under our new drug discovery program, we have needed this intermediate (**6**) in bulk quantity. Synthesis of this compound however involves the use of expensive L-prolinamide (**5**) [6,24–25] (Scheme 1), preparation of which in turn requires an *N*-protection/deprotection strategy. Moreover, the overall yield of isolated product from **5** was only 52% (Scheme 1). It has therefore become necessary for us to develop an alternative method for rapid access to this intermediate in good yield. Herein we report a practical and convenient method for the synthesis of compound **6** using readily available reagents and starting materials.

## Results and Discussion

**Scheme 2:**
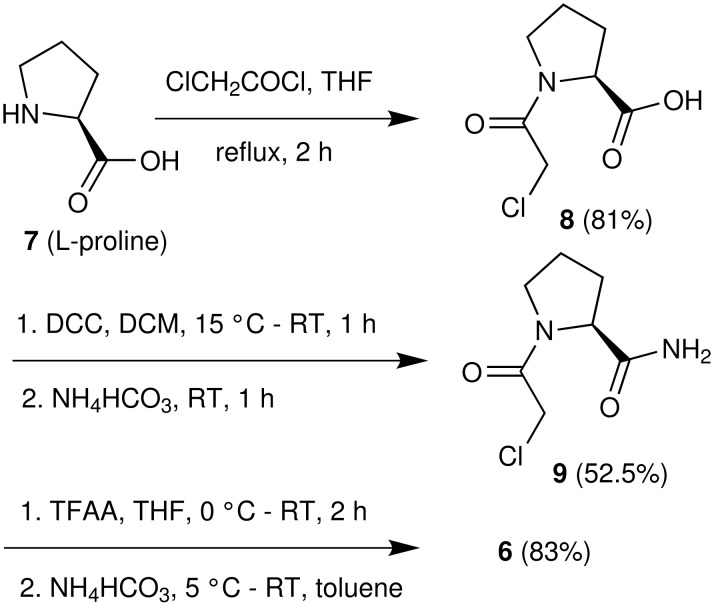
Synthesis of (*S*)-1-(2-chloroacetyl)pyrrolidine-2-carbonitrile (**6**).

In our strategy, we decided to use L-proline in place of L-prolinamide because of its easy availability. Additionally, we anticipated that the chloroacetyl group (which is also a part of the target intermediate) might play the role of a protecting group so that the use of an additional protecting group and its removal (i.e. deprotection) can be avoided. Thus L-proline (**7**) was *N*-acylated with chloroacetyl chloride in refluxing THF to afford 1-(2-chloroacetyl)pyrrolidine-2-carboxylic acid (**8**) (Scheme 2). While preparation of this compound has been reported earlier [26], we encountered several difficulties when following the reported process, the major one being the longer reaction time (48 h) at a low temperature (−20 °C). Nevertheless, we observed that *N*-acylation of L-proline proceeds faster in THF at an elevated temperature. Thus changing the solvent from MeCN [26] to THF and conducting the reaction at reflux we were able to prepare compound **8** in 81% yield within 2 h. Next, we planned to convert the carboxylic acid moiety of compound **8** to the amide which subsequently could afford the desired cyano derivative **6**. Accordingly, several attempts were made to prepare the required amide using standard procedures documented well in the literature. Thus, the acid **8** was treated with a number of reagents [e.g. (i) ethyl chloroformate / Et_3_N or (ii) isobutyl chloroformate / Et_3_N or (iii) SOCl_2 _or oxalyl chloride] separately followed by aqueous ammonia in the same pot. However, in all these cases the desired amide was isolated in poor yield partially due to the loss of product during aqueous work-up. The amide was found to be soluble in water. Unsatisfactory yields and complicated work up prompted us to develop an alternative method avoiding the use of aqueous media or aqueous work up.

With this objective the compound **8** was treated with dicyclohexylcarbodiimide (DCC) at ambient temperature in dichloromethane followed by ammonium bicarbonate (Scheme 2). Encouragingly, the reaction proceeded well as indicated by TLC. After filtering the reaction mass, the filtrate was concentrated and the crude residue isolated was purified by crystallization followed by column chromatography to afford the desired amide **9** in 52% yield without exposure to aqueous conditions. To prepare the target cyano pyrrolidine **6**, which was also found to be soluble in water, a solution of amide **9** in THF was treated with trifluoroacetic anhydride. After completion of the reaction the side product trifluoroacetic acid was neutralized by ammonium bicarbonate and the desired product **6** was isolated (HPLC purity 99.25%) from the toluene extract without aqueous work up. Thus compound **6** was prepared in 83% yield from amide **9** (~30% overall yield from L-proline **7**). *N*-Acylproline derivatives are known to exist as a mixture of *cis*- and *trans*-amide rotamers in solution. The same has been observed in case of *N*-acyl-2-cyanopyrrolidines [6]. Thus, as indicated by ^1^H NMR spectra the protons at C-2 position of the pyrrolidine ring (and of the CH_2_Cl group) tended to appear separately in the corresponding spectra (Figure 3, see Supporting Information File 1). The presence of rotamers was also evident in the ^13^C NMR spectra (Figure 4, see Supporting Information File 1). Based on ^1^H NMR data it was possible to calculate the ratio of rotamers present in the solution. The spectral data of compound **6** were identical to the reported data [6].

**Scheme 3:**
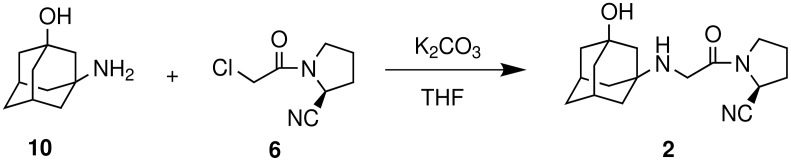
Preparation of Vildagliptin (**2**).

Having prepared the key intermediate **6** successfully we treated it with a variety of aliphatic and aromatic amines to generate a library of compounds for biological screening. Moreover, the DPP-IV inhibitor Vildagliptin or (2*S*)-{[(3-hydroxyadamantan-1-yl)amino]acetyl}-pyrrolidine-2-carbonitrile (**2**) was prepared in 50% yield by reacting the intermediate **6** with 3-hydroxy-1-aminoadamantane (**10**) in the presence of K_2_CO_3_ at room temperature according to the known procedure (Scheme 3) [6].

## Conclusion

In conclusion, we have demonstrated an alternative and practical route for the synthesis of (*S*)-1-(2-chloroacetyl)pyrrolidine-2-carbonitrile, a key intermediate for the synthesis of DPP-IV inhibitors, starting from less expensive and readily available L-proline. In comparison to the earlier routes the present process involves neither *N*-protection / deprotection strategy nor a complicated isolation method. The utility of this process has been demonstrated in the synthesis of Vildagliptin, a potent, selective and orally bio-available DPP-IV inhibitor currently waiting for FDA approval. We expect that this process would find applications in the design and synthesis of novel DPP-IV inhibitors for the potential treatment of type-II diabetes.

## Experimental

### General methods

All the compounds synthesized were characterized by NMR, IR and MS spectra. ^1^H NMR spectra were recorded on a Bruker Avance 300 spectrometer (300 MHz). Electrospray (ES+) mass spectra were acquired on an ion trap mass spectrometer. HPLC purity was determined with an Alliance Waters instrument (model 2695) equipped with a Hypersil BDS C18 column, 250 × 4.6 mm, 5 μm and a 2996 UV detector [buffer: 1000 mL water of pH 3.0 with TFA, mobile phase A, buffer : acetonitrile (95:5), mobile phase B, buffer : acetonitrile (10:90), 55 min, 220 nm, 1.0 mL/min]. Elemental analyses were performed on a Thermo Finnigan analyzer and were within 0.4% of the theoretical values.

### Preparation of (*S*) 1-(2-Chloroacetyl)pyrrolidine-2-carboxylic acid (8)

To a suspension of L-proline (20.0 g, 0.174 mol) in THF (200 mL) was added chloroacetyl chloride (19.7 ml, 0.261 mol) at room temperature and the reaction mixture was refluxed for 2 h. After completion of the reaction, the mixture was cooled to room temperature, diluted with water (20 mL) and stirred for 20 min. Saturated brine (20 mL) and ethyl acetate (200 mL) were added and the organic layer was collected. The aqueous layer was re-extracted with ethyl acetate (2 × 50 mL). The combined organic extracts were dried over anhydrous Na_2_SO_4_ and concentrated under vacuum. The semisolid residue was stirred in diisopropyl ether (100 mL) for 0.5 h at room temperature and the mixture was then cooled to 0 °C for 1 h. The precipitated crystalline white solid was filtered, washed with cold diisopropyl ether and dried at 40 °C under vacuum to afford compound **8** (27.0 g, 81.1%); mp 108–110.9 °C; [α]_D_^25^ −106.2 (*c* 1.00, CHCl_3_); IR (KBr, cm^−1^): 3420, 3050, 2989, 2940, 2811, 1723, 1611, 1476, 1463; ^1^H NMR (300 MHz, CDCl_3_) δ 2.0–2.4 (m, 4H), 3.59–3.74 (m, 2H), 4.0–4.2 (m, 0.2H, C*H*_2_Cl), 4.1 (s, 1.8H, C*H*_2_Cl), 4.6 (m, 1H, C*H*COOH), 7.1 (bs, 1H, COOH); ^13^C NMR (75 MHz, CDCl_3_) δ 22.2, 24.8, 28.7, 31.2, 41.6, 41.7, 47.2, 47.3, 59.3, 59.5, 166.1, 166.4, 174.8, 174.9; m/z 192.1 [M+1]; Anal. Calcd for C_7_H_10_ClNO_3_: C, 43.88; H, 5.26; N, 7.31. Found: C, 43.25; H, 4.91; N, 6.98.

### Preparation of (*S*) 1-(2-chloroacetyl)pyrrolidine-2-carboxamide (9)

To a solution of compound **8** (10.0 g, 0.052 mol) in dichloromethane (200 mL) was added slowly a solution of dicyclohexylcarbodiimide (10.8 g, 0.052 mol) in dichloromethane at 10–15 °C (duration 5.0 min) and the mixture was stirred at room temperature for 1 h. To this was added ammonium bicarbonate (41.2 g, 0.522 mol) at precipitated and the mixture was stirred for 1 h. The reaction was monitored by TLC (5% MeOH-CHCl_3_, anisaldehyde & I_2_). After completion of the reaction, the mixture was filtered and the residue was washed with DCM. The filtrates were collected, combined and concentrated under vacuum. The resulting gummy mass was suspended in THF (30 mL) under stirring and diisopropyl ether (120 mL) was slowly added dropwise over 15 min. The mixture was then cooled to 0 °C and allowed to stand at this temperature for 1 h. The resulting crystalline white solid was filtered, washed with diisopropyl ether and dried under vacuum at 40 °C to afford the crude product **9** (6.3 g, 63.6%). This was then purified by column chromatography (eluting solvent: 2% MeOH/CHCl_3_) followed by crystallization with diisopropyl ether to afford the pure crystalline compound **9** (5.2 g, 52.5%). mp 133–137 °C; [α]_D_^25^ −163.3 (*c* 1.00, CHCl_3_); IR (KBr, cm^−1^): 3383, 3156, 2982, 2942, 2885, 2765; ^1^H NMR (300 MHz, CDCl_3_+CD_3_OD) 2.0–2.2 (m, 4H), 3.55–3.75 (m, 2H), 4.06 (m, 0.4H, C*H*_2_Cl), 4.16 (m, 1.6H, C*H*_2_Cl), 4.47 (m, 1H, C*H*CONH_2_); ^13^C NMR (75 MHz, CDCl_3_) δ 24.9, 28.1, 42.0, 47.4, 60.1, 166.3, 173.3; m/z 191.1 [M+1]. Anal. Calcd for C_7_H_11_ClN_2_O_2_: C, 44.11; H, 5.82; N, 14.7. Found: C, 43.96; H, 5.21; N, 14.18.

### Preparation of (*S*) 1-(2-chloroacetyl)pyrrolidine-2-carbonitrile (6)

To a suspension of amide **9** (4.0 g, 0.0209 mol) in THF (40 mL) was added trifluoroacetic anhydride (4.4 mL, 0.0315 mol) at 0–5 °C and the reaction mixture was then stirred at room temperature for 2 h. The reaction was monitored by TLC (5% MeOH/CHCl_3_, anisaldehyde active). To this mixture was added portion wise (over 5 min) ammonium bicarbonate (12.4 g, 0.1573 mol) maintaining the temperature of the mixture at 5–10 °C. The mixture was stirred at room temperature for 45 min and then concentrated under vacuum at 40 °C. The residue was stirred in toluene (60 mL) at room temperature for 1.0 h. After filtration, the filtrate was concentrated under vacuum at 40 °C to afford an oily mass which was stirred in hexane (20 mL) at room temperature for 30 min. The mixture was cooled to 0–5 °C and allowed to stand at the same temperature for 30 min. The resulting crystalline solid was filtered and washed with cold hexane to give the target compound **6** (3.0 g, yield 83%); HPLC Purity: 99.25%; mp 52–53 °C (lit [6] 53–57 °C); IR (KBr-, cm^-1^): 3304, 2992, 2953, 2888, 2242, 1662, 1424; ^1^H NMR (300 MHz, CDCl_3_) δ (4:1 mixture of trans/cis amide rotamers) 2.1–2.4 (m, 4H), 3.56–3.64 (m, 1H), 3.69–3.76 (m, 1H), 4.02–4.21 (m, 0.4H, C*H*_2_Cl), 4.06 (s, 1.6H, C*H*_2_Cl), 4.76 (m, 0.8H, C*H*CN), 4.86 (m, 0.2H, C*H*CN); ^13^C NMR (75 MHz, CDCl_3_) δ 22.7, 24.6, 25.1, 29.9, 32.4, 41.6, 46.4, 46.7, 46.9, 47.0, 117.8, 164.7, 165.2; m/z 173.1 [M+1]; Anal. Calcd for C_7_H_9_ClN_2_O: C, 48.71; H, 5.26; N, 16.23. Found: C, 47.98; H, 4.96; N, 16.01.

## Supporting Information

File 1 ^1^H and ^13^C NMR spectra of compound **6** in CDCl_3_.
