# Supplementary material for: Synthesis of (S)-1-(2-chloroacetyl)pyrrolidine-2-carbonitrile: A key intermediate for dipeptidyl peptidase IV inhibitors
Source: Beilstein J Org Chem. 2008 Jun 12;4:20. doi: 10.3762/bjoc.4.20 (PMC2486489; doi:10.3762/bjoc.4.20)
Supplement: File 1 — 1H and 13C NMR spectra of compound 6 in CDCl3. [file Beilstein_J_Org_Chem-04-20-s001.doc]

# Supporting Information

**Synthesis of (*S*)-1-(2-chloroacetyl)pyrrolidine-2-carbonitrile: A key intermediate for dipeptidyl peptidase IV inhibitors**

Santosh Kumar Singh*, Narendra Manne and Manojit Pal*

Address: New Drug Discovery, Matrix Laboratories Limited, Anrich Industrial Estate, Bollaram, Jinnaram Mandal, Medak Dist., Andhra Pradesh, India-502 325

* Corresponding authors

**Synthesis of (*S*)-1-(2-chloroacetyl)pyrrolidine-2-carbonitrile supporting info.**

^1^H and ^13^C NMR spectra of compound **6**


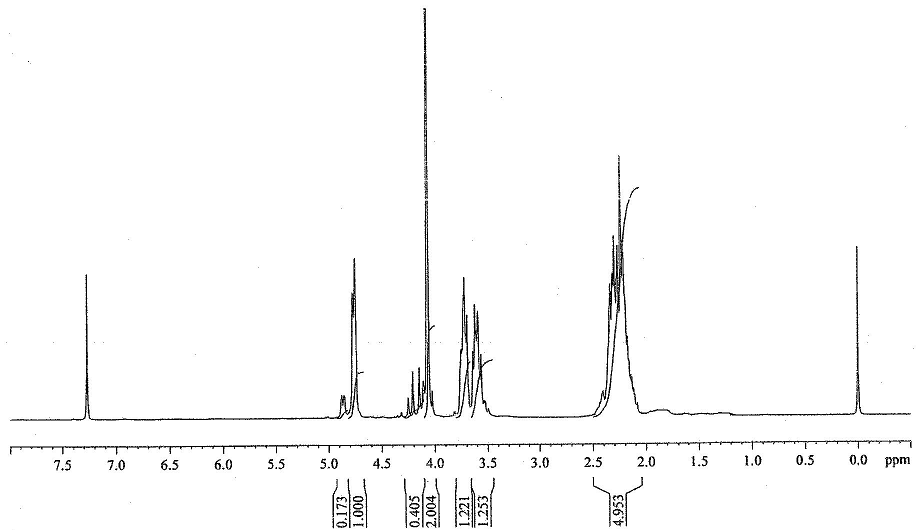


**Figure 3:** ^1^H NMR spectrum of compound **6** in CDCl_3_.


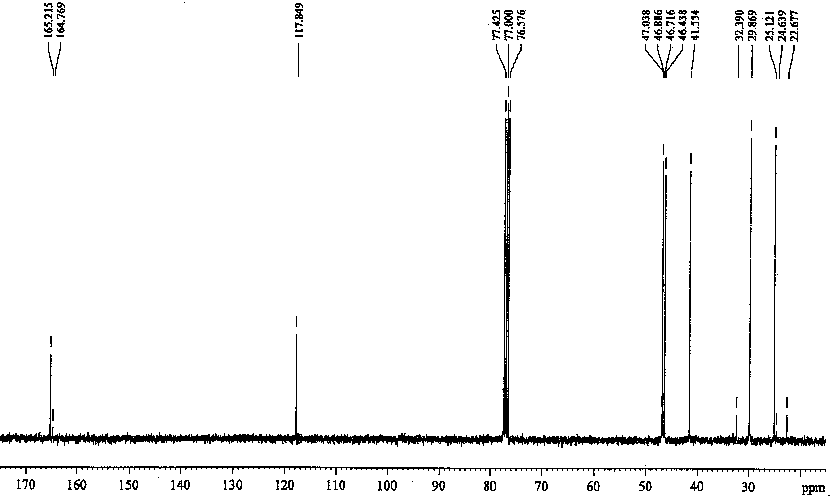


**Figure 4:** ^13^C NMR spectrum of compound **6** in CDCl_3_.
